# Histological, behavioural and flow cytometric datasets relating to acute ischaemic stroke in young, aged and ApoE^−/−^ mice in the presence and absence of immunomodulation with fingolimod

**DOI:** 10.1016/j.dib.2021.107146

**Published:** 2021-05-15

**Authors:** Andrea C. Diaz Diaz, Kyle Malone, Jennifer A. Shearer, Anne C. Moore, Christian Waeber

**Affiliations:** a Department of Pharmacology and Therapeutics, Western Gateway Building, University College Cork, Cork, Ireland; b School of Pharmacy, University College Cork, Cork, Ireland; c School of Biochemistry and Cell Biology, University College Cork, Cork, Ireland

**Keywords:** Fingolimod, Stroke, Ischaemia, Inflammation, T cells, Lymphocytes, Neuroinflammation

## Abstract

In this work, the sphingosine-1-phosphate receptor modulator fingolimod was assessed as a preclinical candidate for the treatment of acute ischaemic stroke according to the Stroke Therapy Academic Industry Roundtable (STAIR) preclinical recommendations. Young (15–17 weeks), aged (72–73 weeks), and ApoE^−/-^ mice (20–21 weeks) fed a high fat diet (all C57BL/6 mice) underwent permanent electrocoagulation of the left middle cerebral artery. Mice received either saline or fingolimod (0.5 mg/kg or 1 mg/kg) at 2-, 24-, and 48-hours post-ischaemia via intraperitoneal (i.p.) injection. Another cohort of young mice (8–9 and 17–19 weeks) received short-term (5 days) or long-term (10 days) fingolimod (0.5 mg/kg) treatment in a treatment duration study. For young, aged, and ApoE^−/-^ mice, motor behavioural tests (cylinder and grid-walking) were performed at days 0, 3, and 7 post-ischaemia to evaluate neurobehavioural recovery. In the treatment duration study, the grid-walking test was performed at days 0, 2, 5 and 10 post-ischaemia. Brain tissue sections were stained with haematoxylin and eosin (H&E), and NeuN to quantify tissue damage. Flow cytometry was used to quantify T cell populations in blood, spleen, and lymph nodes. The data presented in this article improves our understanding of the potential neuroprotective and immunomodulatory effects of fingolimod in a mouse model of brain ischaemia. Such data may be significant in the design of future preclinical and clinical stroke studies for fingolimod.

**Specifications Table**

**Table:** 

Subject	Neuroscience: General
Specific subject area	Histological and behavioural outcomes in a mouse model of acute ischaemic stroke, effects of co-morbidity on functional outcomes post-stroke, effects of stroke on immune cells, immunomodulatory drugs for the treatment of stroke.
Type of data	Table (within repository file)
How data were acquired	Flow cytometer: LSRII (Becton Dickinson) FACS data analysis software: FlowJo (v10) Microscope: Olympus BX51 Image Analysis Software: ImageJ Video camera: Canon Legria HFR706 Statistical Analysis/Figure Generation: GraphPad Prism 8.0
Data format	Raw Analysed
Parameters for data collection	Young, aged, and ApoE^−/−^ mice fed a high fat diet (all C57BL/6 mice) underwent permanent electrocoagulation of the left middle cerebral artery. Mice received saline or fingolimod (0.5 mg/kg or 1 mg/kg) at 2-, 24-, and 48-hours post-ischaemia via intraperitoneal (i.p.) injection. Another cohort of young mice received either saline or short-term (5 days) or long-term (10 days) fingolimod (0.5 mg/kg) treatment.
Description of data collection	All animals were evaluated daily for weight, behavioural, and neurological recovery from baseline through to the end of the experimental timeline. In young, aged, and ApoE^−/−^ mice, neurobehavioural recovery was evaluated at days 0, 3 and 7 post-ischaemia using cylinder and grid-walking tests. In the treatment duration study, neurobehavioural recovery was assessed using the grid-walking test at days 0, 2, 5, and 10 post-ischaemia. Brain tissue damage was determined from haematoxylin and eosin (H&E), and NeuN stained sections. Flow cytometry was used to quantify immune cell frequencies and total cell counts in blood, spleen, and lymph nodes. Immunohistochemistry was used to quantify immune cell infiltration into the ischaemic brain.
Data source location	School of Pharmacy, University College Cork, Cork, Ireland.
Data accessibility	Repository name: Mendeley Data Direct URL to data: http://dx.doi.org/10.17632/ctyx59p5rh.1
Related research article	Malone K, Diaz Diaz AC, Shearer JA, Moore AC, Waeber C. The Effect of Fingolimod on Regulatory T Cells in a Mouse Model of Brain Ischaemia. Journal of Neuroinflammation; 2021.

## Value of the Data

• The data included herein provide a comprehensive insight into the effect of permanent brain ischaemia on histological, behavioural, and immunological outcomes in young mice as well as in models of stroke co-morbidity (age, hypercholesteremia). Such data are useful for any researcher involved in the study of stroke pathophysiology, stroke recovery, post-stroke changes in immune function, and treatment with the immunomodulator fingolimod.
• While previous research has investigated the effect of immunomodulatory therapies in rodent models of brain ischaemia, many aspects of the STAIR preclinical recommendations (adequate dose-response curve, defined treatment duration, blinded studies, and inclusion of aged/co-morbid animals) remain unaddressed [2],[3]. These data will not only be of significant interest to researchers involved in the development of ischaemic stroke therapies but also all those interested in rigorous and well-powered preclinical trials in stroke.
• The behavioural and neurological outcome data could be useful for researchers conducting effect size calculations ahead of grant applications or pilot studies.
• The flow cytometric data may be of interest to researchers interested in correlating levels of immune cells post-stroke with clinical outcomes, or to those engaged in data mining or the development of machine learning algorithms.
• Fingolimod is one of the most compelling drug candidates characterised in preclinical stroke studies to date [1]. But despite proving effective in rodent models of the disease, the optimal dose and treatment duration have not been elucidated. Likewise, the effect of the drug in models of stroke co-morbidity (age, hypercholesteremia) remains unknown. Exact details of the mechanism of action of fingolimod in stroke also remain unclear. This dataset highlights the neuroprotective and immunomodulatory effects of fingolimod in a mouse model of brain ischaemia. Data relating to optimal dose, differing treatment durations, and outcomes in mice with stroke co-morbidities are also outlined.

## 1 Data Description

“Dose Response DB” contains all raw data associated with the study of the effect of 0.5 mg/kg vs. 1 mg/kg fingolimod treatment (vs. saline control) in post-ischaemic C57BL/6 mice. The “Mouse Registry” sheet details mouse IDs, mouse age, and full details of pMCAO procedure. The “Group” sheet shows the treatments which were randomly allocated to each mouse. The “Score Sheets” tab details the daily weights of mice post-ischaemia, as well as scores related to appearance, behaviour and neurological function. The “Cylinder data” sheet shows the raw scores of the cylinder behavioural test at days 0, 3, and 7 post-ischaemia. The “Grid data” sheet shows the raw scores of the grid behavioural test at days 0, 3, and 7 post-ischaemia. The “Histology” sheet details brain lesion sizes (mm^3^) and hemispheric volumes (mm^3^) as determined by H&E and NeuN staining. Finally, the “Flow Cytometry” sheet lists the cell frequencies and counts obtained for CD3+, CD4+, CD8+, and Treg cells in spleen, cervical lymph nodes, inguinal lymph nodes, and blood.

“Aged Study DB” contains all raw data associated with the study of the effect of 0.5 mg/kg fingolimod treatment vs. saline control in post-ischaemic aged C57BL/6 mice. The “Mouse Registry” sheet details mouse IDs, mouse age, and full details of pMCAO procedure. The “Group” sheet shows the treatments which were randomly allocated to each mouse. The “Score Sheets” tab details the daily weights of mice post-ischaemia, as well as scores related to appearance, behaviour and neurological function. The “Cylinder data” sheet shows the raw scores of the cylinder behavioural test at days 0, 3, and 7 post-ischaemia. The “Grid data” sheet shows the raw scores of the grid behavioural test at days 0, 3, and 7 post-ischaemia. The “Histology” sheet details brain lesion sizes (mm^3^) and hemispheric volumes (mm^3^) as determined by H&E and NeuN staining. Finally, the “Flow Cytometry” sheet lists the cell frequencies and counts obtained for CD3+, CD4+, CD8+, and Treg cells in spleen, cervical lymph nodes, inguinal lymph nodes, and blood.

“ApoE DB” contains all raw data associated with the study of the effect of 0.5 mg/kg fingolimod treatment vs. saline control in post-ischaemic ApoE^−/−^ mice. The “Mouse Registry” sheet details mouse IDs, mouse age, and full details of pMCAO procedure. The “HFD Weights” sheet lists the weekly weights of mice during the 12 weeks that they received the high-fat diet. The “Group” sheet shows the treatments which were randomly allocated to each mouse. The “Score Sheets” tab details the daily weights of mice post-ischaemia, as well as scores related to appearance, behaviour and neurological function. The “Cylinder data” sheet shows the raw scores of the cylinder behavioural test at days 0, 3 and 7 post-ischaemia. The “Grid data” sheet shows the raw scores of the grid behavioural test at days 0, 3 and 7 post-ischaemia. The “Histology” sheet details brain lesion sizes (mm^3^) and hemispheric volumes (mm^3^) as determined by H&E and NeuN staining. Finally, the “Flow Cytometry” sheet lists the cell frequencies and counts obtained for CD3+, CD4+, CD8+, and Treg cells in spleen, cervical lymph nodes, inguinal lymph nodes, and blood.

“Duration Study DB” contains all raw data associated with the study of the effect of short-term (5 days) vs. long-term (10 days) treatment with 0.5 mg/kg fingolimod compared to saline control in post-ischaemic C57BL/6 mice. The “Mouse Registry” sheet details mouse IDs, mouse age, and full details of pMCAO procedure. The “Group” sheet shows the treatments which were randomly allocated to each mouse. The “Score Sheets” tab details the daily weights of mice post-ischaemia, as well as scores related to appearance, behaviour and neurological function. The “Grid data” sheet shows the raw scores of the grid behavioural test at days 0, 2, 5, and 10 post-ischaemia. The “Histology” sheet details brain lesion sizes (mm^3^) and hemispheric volumes (mm^3^) as determined by H&E and NeuN staining. Finally, the “Flow Cytometry” sheet lists the cell frequencies and counts obtained for CD3+, CD4+, CD8+, and Treg cells in spleen, cervical lymph nodes, inguinal lymph nodes, and blood.

“Naive DB” contains all raw data associated with the study of the effect of brain ischaemia in both young and aged C57BL/6 mice. The “Mouse Registry” sheet details mouse IDs, mouse age, and full details of pMCAO procedure. The “Group” sheet shows the age and sex of mice, as well the allocation to naïve, sham, or pMCAO groups. The “Score Sheets” tab details the daily weights of mice post-ischaemia, as well as scores related to appearance, behaviour and neurological function. The “Cylinder Data” sheet shows the raw scores of the cylinder behavioural test at days 0, 3, and 7 post-ischaemia. The “Histology” sheet details brain lesion sizes and hemisphere volumes (mm^3^) as determined by H&E and NeuN staining (data to follow). Finally, the “Flow Cytometry” sheet lists the cell frequencies and counts obtained for CD3+, CD4+, CD8+, and Treg cells in spleen, cervical lymph nodes, inguinal lymph nodes, and blood.

**Fig. 1:**
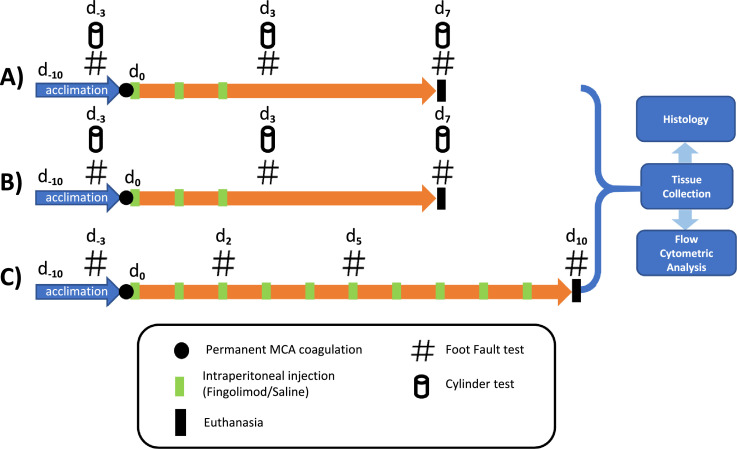
**Data in Brief Fingolimod Study Flowcharts.** **A)** Dose Response study: Young mice (15–17 weeks) underwent permanent electrocoagulation of the left middle cerebral artery. Mice received either saline or fingolimod (0.5 mg/kg or 1 mg/kg) at 2-, 24-, and 48-hours post-ischaemia via intraperitoneal (i.p.) injection. Cylinder and grid-walking neurobehavioural tests were performed at days 3 and 7 post-ischaemia. **B)** Aged and ApoE studies: Aged mice (72–73 weeks) and ApoE^−/−^ (20–21 weeks) underwent permanent electrocoagulation of the left middle cerebral artery. Mice received either saline or fingolimod (0.5 mg/kg) at 2-, 24-, and 48-hours post-ischaemia via intraperitoneal (i.p.) injection. Cylinder and grid-walking neurobehavioural tests were performed at days 3 and 7 post-ischaemia. **C)** Duration study: Young mice (8–9 and 17–19 weeks) underwent permanent electrocoagulation of the left middle cerebral artery. Mice received short-term (5 days) or long-term (10 days) fingolimod (0.5 mg/kg) via intraperitoneal injection. Grid-walking neurobehavioural tests were performed at days 2, 5 and 10 post-ischaemia. In all studies, brain lesions were quantified via haematoxylin and eosin (H&E) and NeuN staining. T cell populations in blood and secondary lymphoid organs were quantified via flow cytometry.

Fig. 1 depicts the study flowcharts for the studies involving fingolimod treatment (Dose Response, Aged, ApoE, Duration) including dosing schedule, behavioural testing, and outputs post-tissue collection.

**Fig. 2:**
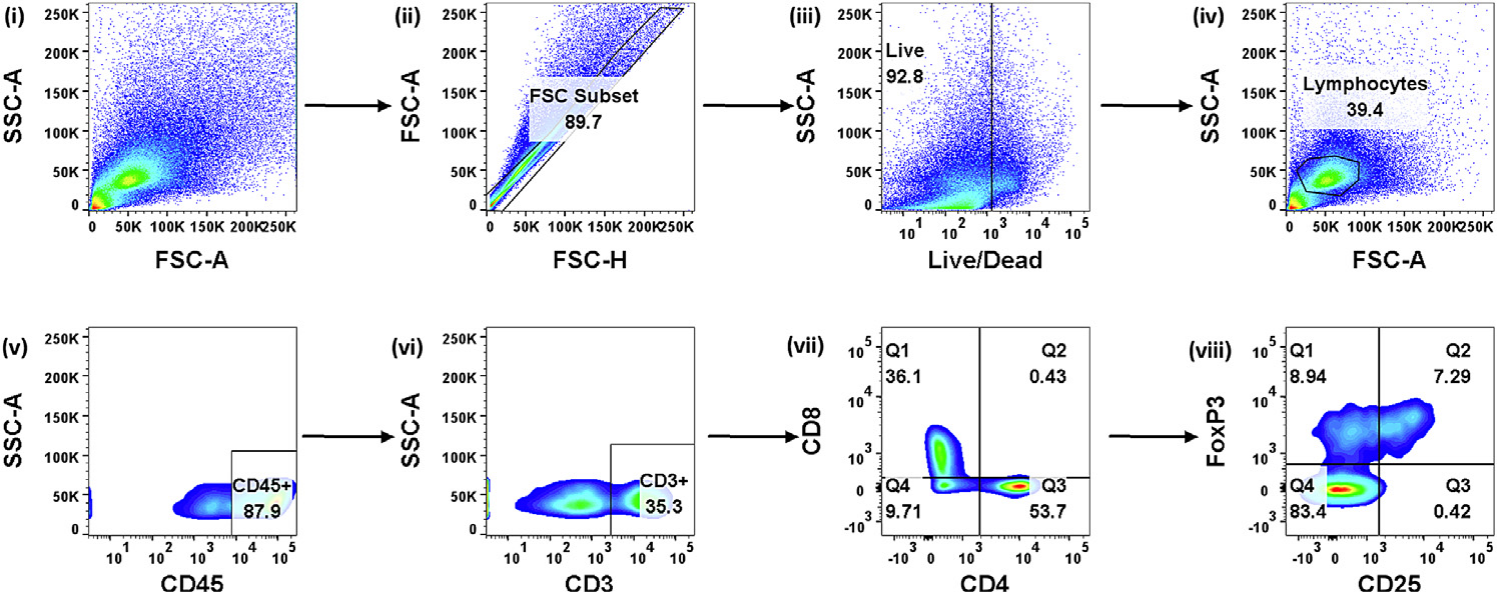
Gating strategy for determination of T cell subsets in spleen. From an initial population, doublets (ii) and dead cells (iii) are first excluded. From total lymphocytes (iv), CD45^hi^ cells are selected. CD3+ T cells (vi) are subdivided into CD4+ and CD8+ compartments (vii). Within the CD4+ pool, CD25 and FoxP3 are then used to determine Tregs.

Fig. 2 depicts the gating strategy employed for the flow cytometric determination of CD3+, CD4+, CD8+ and Treg cells across spleen, cervical lymph nodes, inguinal lymph nodes, and blood.

## 2 Experimental Design, Materials and Methods

### 2.1 Mice

A total of *n* = 257 mice were included as part of these studies. Mice were sourced from an approved European vendor. Young male C57BL/6JOlaHsd mice were ordered from Envigo, UK, at 6–8 weeks of age. A set of 33 male C57BL/6J aged mice were received from Charles River, UK, at 58 weeks of age. Another set of male and female aged mice were received from Charles River, UK, at 62 and 58–59 weeks of age respectively. Male B6.129P2 APOE/J ordered from Charles River, Italy, were received at 4–5 weeks of aged. The ApoE/J mice were started on a 12 week adjusted calorie diet (TD.88137; Envigo, USA) at 8 weeks of age. Lastly, naïve male and female mice (C57BL/6J) were ordered and received from Charles River, UK, at 8 weeks of age. All of the mice were allowed to acclimatize (≥1 week) prior to any procedure being performed.

Mice were housed in groups of 3–4 in individually ventilated cages. In the specific-pathogen-free facility, mice were exposed to a 12-hour light/12-hour dark cycle and kept at a temperature of 20‑24 °C and a relative humidity of 45‑65%. Mice had ad libitum access to both food and water. They were also provided environmental enrichment (i.e. wooden blocks and cardboard cylinders).

An *a priori* sample size calculation based on a previously published meta-analysis [4] showed that *n* = 16 mice per group were required to detect changes in infarct size (significance level set at α = 0.05, power of 80%). The flow cytometry sample sizes were based on a pilot study that confirmed *n* ≥ 3 per group would be sufficient to detect the expected effect of fingolimod on T cell frequencies in spleen. Where possible, tissue samples from all mice were collected to maximise power and allow for comparison of immunological results with functional outcomes.

The pre-determined exclusion criteria removed mice with serious uncontrollable haemorrhage as well as mice with thermal or physical damage to the cortex from the studies. Mice were randomly allocated to a treatment group using a pseudo-random number generator (randomizer.org) prior to surgery. The allocation remained concealed to the surgeon until treatment was administered.

### 2.2 Ischaemia model

A permanent distal middle cerebral artery occlusion model was performed as previously described [5]. Briefly, mice were anaesthetised with 2 - 2.5% isoflurane for induction and maintained with 1.5 - 2% isoflurane evaporated in O2/N2 (30%/70%). Anaesthetic depth was confirmed by absence of the pedal withdrawal reflex. Body temperature was monitored and maintained at 37 °C with a homeothermic feedback system. The incision area, between the left ear and left eye, was cleared of hair with a depilatory cream (Veet, Reckitt Benckiser). The site was cleaned and sterilised with 70% alcohol and povidone solution in 3 cycles. Bupivacaine was infiltrated (0.5%; 0.1 ml) in the incision site.

An incision (1 cm) was made to expose the temporal muscle. The temporal muscle was retracted to expose the parietal bones where a small craniotomy was performed. Once the bifurcation of the middle cerebral artery was exposed, the dura was removed. The middle cerebral artery, including the branches, were occluded using bipolar electrocoagulation forceps (McPherson 3 1/2″ straight forceps (A842); Bovie Bantam Pro electrosurgical generator (A952); Symmetry Surgical Inc, USA). The middle cerebral artery was nicked to confirm successful occlusion of the artery and prevent reperfusion. The burr hole was covered with bone wax and the incision was closed. Anaesthesia was discontinued and mice were allowed to recover for 30 min in a heated chamber (32 °C) before being returned to their home cage. Mice were monitored daily through use of a scoresheet which recorded weight loss, appearance changes, behaviour, and neurological function.

Mice receiving treatment in the Dose Response, Aged, and ApoE studies were dosed at 2-, 24- and 48-hours post‑ischaemia. Mice in the dose response study received saline, 0.5 mg/kg or 1 mg/kg fingolimod (Novartis Institutes for Biomedical Research, Switzerland) via i.p. injection. Mice in the Aged and ApoE-/- studies received saline or 0.5 mg/kg fingolimod via i.p. injection. The mice in the treatment duration study received daily injection of either (1) saline, (2) 5 days of fingolimod (0.5 mg/kg) followed by 5 days of saline, or (3) 10 days of fingolimod (0.5 mg/kg). A researcher not associated with the surgery prepared treatment solutions (pH 7) for volumes no greater than 250 µl per injection. Sham surgeries only involved the incision and retraction of the temporal muscle.

### 2.3 Neurological deficit evaluation

Behavioural assessment was performed at baseline and at 3 and 7 days post-ischaemia in the dose response, aged and ApoE studies, and 2, 5 and 10 days in the duration study. The test sessions were recorded for quantification at a later date. For the cylinder test, mice were placed in a cylinder (d: 12.5 cm x h: 23.5 cm) where they explored the surface until 20 wall rears were completed. The score was calculated from the number of the independent wall touches with the left (L) or right (R) forepaw, or simultaneous use of both (B) [deficit score = (R-L)/(*R* + *L* + *B*)]. For the grid walking (foot fault) test, mice were placed on a wire grid (25 cm x 35 cm) with 1 cm^2^ openings and encouraged to walk from one end to the other. During analysis, the number of missed contralateral and ipsilateral steps were counted up until the first 100 steps. The deficit score was then calculated as the ratio of contralateral/ipsilateral missed steps.

### 2.4 Tissue collection and processing

At 7 days post-ischaemia (or at 10 days in the treatment duration study), mice were euthanised by anaesthetic overdose (15–30 µL; 200 mg/ml pentobarbital). Blood was collected from the descending aorta and transferred into EDTA-coated tubes (approximately 500 µl). Mice were then perfused transcardially with 20 ml cold phosphate buffered saline (PBS) (P4417; Sigma Aldrich). The brain was isolated, frozen in isopentane (Sigma Aldrich) at ‑42 °C and stored at −20 °C. Brain sections (20 µm) were cut at 500 µm intervals using a cryostat. Slides were stored at −20 °C until further processing. Lymphoid tissues (spleen, cervical and inguinal lymph nodes) were harvested and stored in PBS. The lymphoid tissues were mechanically dissociated in approximately 3 ml sterile DPBS in a sterile 6-well plate using a textured thumb press from a 3 ml syringe plunger. The resulting cell suspensions was passed through a 70 µm cell strainer and collected in a 50 ml conical tube. Both the wells and strainers were rinsed twice with 1X DPBS (D8537; Sigma Aldrich). Cells were re-suspended in 5 ml of 1X red blood cell lysis buffer (430,054; eBioscience) and incubated for 5 min at room temperature. The lysis reaction was stopped by adding 20 ml of 1X PBS. The cell samples were washed twice with 1X PBS and re-suspended in an appropriate volume of PBS. The cells were counted using trypan blue to determine total cell concentration and viability.

### 2.5 Flow cytometric analysis

The antibodies used were sourced from eBioscience unless otherwise stated. Cell suspension samples were incubated for 5 min with anti-mouse CD16/CD32 (50 µl, 1:100, Clone 93; 14016182). The respective cell suspensions were then stained for anti-mouse CD45 (30-F11, 1:100, PerCP-CY5.5; 45045182), CD3 (1:100, 145–2C11, PE-Cy7, 14003182), CD4 (1:800, RM4–5, FITC, 11004282), CD8 (1:100, 5H10, Pacific Blue, MCD0828), and CD25 (1:100, PC61.5, APC, 17025182). A live/dead stain (1:10,000, Fixable Viability Dye eFluor 780, 65086514) was also added to each sample. Samples were incubated in the dark for 30 min at 2‑8 °C. Post‑incubation, the samples were washed, fixed, permeabilised, and stained intracellularly with anti-mouse FoxP3 (1:100, FJK-16 s, 12577382), in accordance with the instructions provided with the Mouse Regulatory T Cell Staining Kit #1 (88811140).

For flow cytometric analysis the samples were re‑suspended in 400 µl of PBS and analysed using an LSRII flow cytometer (Becton Dickinson). Compensation control was set using BD CompBead Anti-Rat/Anti-Hamster Particles Set (552845, Becton Dickinson). Data were analysed using FlowJo (v10) according to the following gating strategy outlined in Fig. 2. Gates were set according to unstained samples and fluorescent minus one controls. Absolute cell counts for all tissues were calculated in accordance with the instructions provided with the CountBright Absolute Counting Beads (C36950; Molecular Probes).

### 2.6 Haematoxylin and eosin staining

Slides were air dried overnight, fixed in 4% formalin for 5 min, and rehydrated in a series of graded ethanol (100%, 95% and 70%) followed by distilled water for 2 min each. Slides were immersed in Mayer's haematoxylin solution (Sigma Aldrich) for 4 min, rinsed in water to remove excess stain and then transferred to 0.25% Eosin Y solution for 1 min. Finally, slides were dehydrated sequentially in a series of graded ethanol (70%, 90% and 100%) for 2 min each and washed twice in histochoice (H2779; Sigma Aldrich) for 2 min. Slides were mounted with Permount (SP15–500, Fisher Scientific).

### 2.7 NeuN immunohistochemistry

Slides were air dried and fixed for 20 min in methanol (Sigma Aldrich) at −20 °C. Endogenous peroxidase activity was blocked by incubation in cold 3% hydrogen peroxide/methanol solution for 10 min. Non‑specific staining was blocked for 1 hour with 5% normal goat serum (S-1000, Vector Laboratories) in 0.3% Triton-X in Tris buffered saline (TBS) at room temperature. Slides were incubated with anti-NeuN antibody (1:1500; ab177487; Abcam) for 1 hour at room temperature followed by goat anti-rabbit biotinylated secondary antibody (1:250; ab207995; Abcam) for 1 hour at RT. Slides were then washed three times with TBS. Immunoreactivity was visualised by the avidin‑biotin complex method (PK-4000; Vector Laboratories) and developed for 10 min with diaminobenzidine (DAB) (SK-4100; Vector Laboratories). Lastly, the slides were counterstained with eosin and dehydrated in a series of graded ethanol (95% and 100%) for 2 min each. Slides were cleared in histochoice twice for 2 min and then mounted with permount.

Stained sections (H&E and NeuN) were scanned at 3200 dpi on an Epson Perfection V550 scanner. Lesion size and hemispheric volumes were measured on scanned images using ImageJ [6]. The volume (mm^3^) of each region was calculated by multiplying the area of interest (mm^2^) by the distance between sections (0.5 mm).

## Ethics Statement

Animal experiments were carried out in accordance with the European Directive 2010/63/EU, following approval by the Animal Experimentation Ethics Committee of University College Cork and under an authorization issued by the Health Products Regulatory Authority Ireland (license numbers AE19130/P042 and AE19130/P075). The studies were conducted and reported according to the ARRIVE guidelines [7].

## CRediT Author Statement

**Andrea C Diaz Diaz:** Methodology, Validation, Formal Analysis, Investigation, Writing - Original Draft, Writing – review & editing, Visualization; **Kyle Malone:** Methodology, Validation, Formal Analysis, Investigation, Writing – Original Draft, Writing – Review and Editing, Visualization; **Jennifer A Shearer:** Methodology, Validation, Formal Analysis, Investigation, Writing – Review and Editing; **Anne C Moore:** Conceptualization, Methodology, Writing – Review and Editing, Visualization, Supervision, Project Administration, Funding Acquisition; **Christian Waeber:** Conceptualization, Methodology, Writing – Review and Editing, Visualization, Supervision, Project Administration, Funding Acquisition.

## Funding

This work was generously supported by a Government of Ireland Postgraduate Scholarship from the Irish Research Council (grant number GOIPG/2017/431), an HRB Health Research Award (HRA-POR-2015–1236), the Interreg Atlantic Area Programme (EAPA_791/2018, 2019–21), and a Science Foundation Ireland Grant (BIAP2015).

## Declaration of Competing Interest

The authors declare that they have no known competing financial interests or personal relationships which have or could be perceived to have influenced the work reported in this article.
